# Characterisation of a Vitrocell® VC 10 *in vitro* smoke exposure system using dose tools and biological analysis

**DOI:** 10.1186/1752-153X-7-146

**Published:** 2013-09-03

**Authors:** David Thorne, Joanne Kilford, Rebecca Payne, Jason Adamson, Ken Scott, Annette Dalrymple, Clive Meredith, Deborah Dillon

**Affiliations:** 1 British American Tobacco, Group R&D, Southampton, Hampshire SO15 8TL, UK; 2 Covance Laboratories Ltd, Otley Road, Harrogate, North Yorkshire HG3 1PY, UK

**Keywords:** Dosimetry, Particle deposition, QCM, Quartz crystal microbalance, CO, In vitro, Whole smoke, Vitrocell®

## Abstract

**Background:**

The development of whole smoke exposure systems have been driven by the fact that traditional smoke exposure techniques are based on the particulate phase of tobacco smoke and not the complete smoke aerosol. To overcome these challenges in this study, we used a Vitrocell® VC 10 whole smoke exposure system. For characterisation purposes, we determined smoke deposition in relationship to airflow (L/min), regional smoke deposition within the linear exposure module, vapour phase dilution using a known smoke marker (carbon monoxide) and finally assessed biological responses using two independent biological systems, the Ames and Neutral Red uptake (NRU) assay.

**Results:**

Smoke dilution correlates with particulate deposition (R^2^ = 0.97) and CO concentration (R^2^ = 0.98). Regional deposition analysis within the linear exposure chamber showed no statistical difference in deposited mass across the chamber at any airflows tested. Biological analysis showed consistent responses and positive correlations with deposited mass for both the Ames (R^2^ = 0.76) and NRU (R^2^ = 0.84) assays.

**Conclusions:**

We conclude that in our study, under the experimental conditions tested, the VC 10 can produce stable tobacco smoke dilutions, as demonstrated by particulate deposition, measured vapour phase smoke marker delivery and biological responses from two independent *in vitro* test systems.

## Background

The association between tobacco smoke and disease is widely understood [1-3] however, many of the disease mechanisms that follow tobacco smoke exposure are not. This is particularly driven by the fact that cigarette smoke is a complex aerosol consisting of approximately 5600 chemicals [4], distributed between the vapour and particulate phases. The vapour phase is the majority fraction, between 90-95%, whereas the particulate phase makes up only 5-10% by weight [5]. The particulate fraction is mostly made up of phenols, esters, alkaloid derivatives, terpenoids, alkanes, aldehydes and ketones, acids, alcohols, nicotine and water. The vapour phase consists of hydrocarbons, aldehydes and ketones, nitriles, heterocyclics, alcohols, acids, esters, hydrogen, helium, nitrogen, carbon monoxide and dioxide and oxygen. Distributed and partitioning unevenly between these two fractions are biologically active chemicals, which have known toxicological properties [6-8].

**Figure 1:**
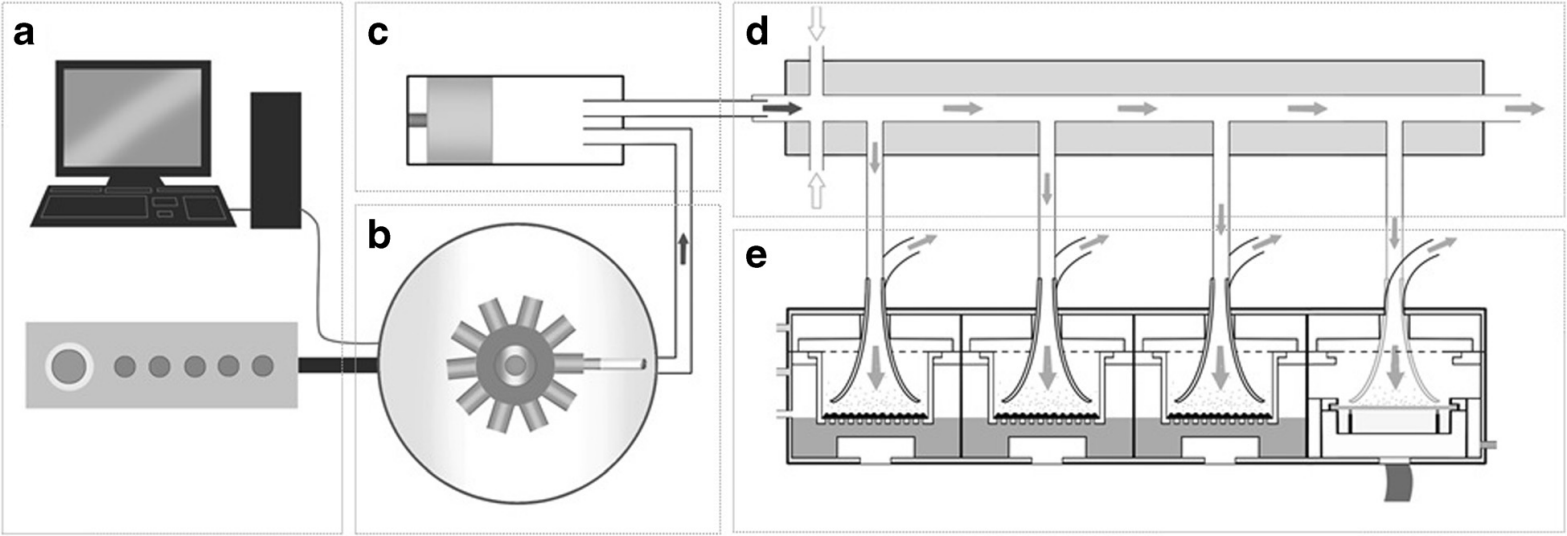
**A schematic representation of the major components of the Vitrocell® VC 10 smoke exposure system. [a]** Computer, software controller and air-flow controller, which determines the smoking parameters and key machine settings. **[b]** Smoking Robot carousel where cigarettes are loaded and smoked, enclosed within an extraction ventilation hood. **[c]** Piston/syringe which draws and delivers an ISO or Health Canada Intense puff (35 ml or 55 ml) of mainstream cigarette smoke to the smoke dilution system. In our set-up smoke is exhausted to the dilution system over 8 seconds, however this can be adjusted. **[d]** Dilution, transit and delivery of whole smoke occurs in the dilution bar, of which multiple bars can make up the complete dilution system. Continuous diluting air is added perpendicular to the mainstream smoke in the range 0.2-12 L/min and administered to the dilution bar through smoke air jets of 2.0 mm diameter. Airflow rates are set by mass flow meters, which can be upgraded to mass-flow controllers. Flow within the dilution system is continuously transiting through to exhaust. **[e]** Smoke exposure module (Vitrocell® 6/4 CF Stainless Steel module or Vitrocell®-AMES) which holds the Transwells® or agar plates which are maintained at the ALI or AAI. Smoke is sampled from the dilution system into the exposure module via negative pressure applied through a vacuum pump at 5 ml/min/well. Smoke is distributed within the exposure module via the smoke ‘trumpet’ inlets and, due to the linear configuration, each culture insert is isolated receiving an independent sample of smoke from the dilution system. The central islands can be removed and quartz crystal microbalances can be installed into each position or, as shown here, in position 4.

Over the last decade a great deal of focus has been placed on the development of tobacco smoke or ‘whole smoke’ related exposure systems [9-12]. This is partly because traditional exposure techniques tend to focus on the particulate phase of cigarette smoke [13,14] and not the complete aerosol. Traditional techniques include capturing the particulate fraction on a Cambridge filter pad and eluting in dimethyl sulphoxide (DMSO) or bubbling the smoke aerosol through cell culture media or phosphate buffered saline (PBS) to obtain a soluble fraction. For both techniques, once the fraction is obtained and dissolved in its respective solvent, cultured cells can be exposed using submerged exposure conditions. Generating a particulate fraction using these techniques is relatively easy and does not require specialised equipment, ensuring a simple yet reliable compound for testing. Unfortunately, as a result, the full interactions of both phases are not captured or assessed *in vitro*. Furthermore, separating smoke fractions may lead to alterations or chemical changes which may not be representative of the complete smoke aerosol [15]. There are a diverse range of whole smoke exposure systems available ranging from commercial set-ups to bespoke in-house designed and developed exposure systems [16-18]. Commercially available systems include those developed by Borgwaldt [19,20], Burghart [21], CULTEX® [22,23] and Vitrocell® [24]. As yet, no exposure system commercially available or otherwise has been completely characterised or validated and each system has advantages and disadvantages over the next [25]. Irrespective of origin, these systems generally have in common two main components: 1, a smoking machine, which generates, dilutes and delivers cigarette smoke; 2, an exposure chamber which houses the associated biological system often at the air-liquid interface (ALI). Exposure of *in vitro* biological systems to tobacco smoke poses many logistical challenges. Not only does the smoke aerosol have to be generated in a consistent manner but, it has to be delivered evenly to the cell culture system and at a biologically relevant dose. One such whole smoke exposure system is the Vitrocell® VC 10 Smoking Robot (Vitrocell® Systems GmbH, Waldkirch, Germany). The VC 10 is a rotary style smoking machine which has a single syringe that transfers the mainstream cigarette smoke to an independent continuous flow dilution system [24]. Smoke dilution in this system is achieved via turbulent mixing, by adding air perpendicular to the stream of smoke. Smoke dilutions are created by increasing or decreasing the diluting airflow. A vacuum sub-samples smoke from the dilution system into the exposure module, which docks directly under the continuous flow dilution system. Inserts containing cells or a quartz crystal microbalance are then exposed at the ALI or air-agar-interface (AAI) to diluted smoke from separate sample ports under the dilution system (Figure 1).

At present, smoke generation, dilution and deposition in the VC 10 Smoking Robot remains largely undefined and uncharacterised. Therefore, this study assesses the distribution of tobacco smoke, both particulate and vapour phase, combined with biological responses *in vitro* using the VC 10 Smoking Robot in order to characterise the system. To quantify particulate deposition, we used a real-time quartz crystal microbalance (QCM) tool [20,24,26]. Carbon monoxide (CO) concentrations were measured to establish vapour phase dilution characteristics and finally, we used two biological systems, the Neutral Red uptake (NRU) and Ames assay to assess biological responses. Both particulate deposition and vapour phase dilution showed correlations of R^2^ = 0.975 and R^2^ = 0.987 respectively with diluting airflow (L/min). Regional smoke deposition across the linear exposure module showed no statistical difference at any of the airflows tested (0.5-4.0 L/min), demonstrating uniform deposition within the chamber at all positions within this system. Furthermore, real-time deposition data was obtained *in situ* of exposure for both the NRU and Ames assays (1.0-12.0 L/min). Finally, biological data from both assays has been presented as a function of real-time deposited mass obtained concurrently with biological exposure, with associated correlations of R^2^ = 0.84 and R^2^ = 0.76 respectively.

## Results

### Measurement of deposited mass

**Figure 2:**
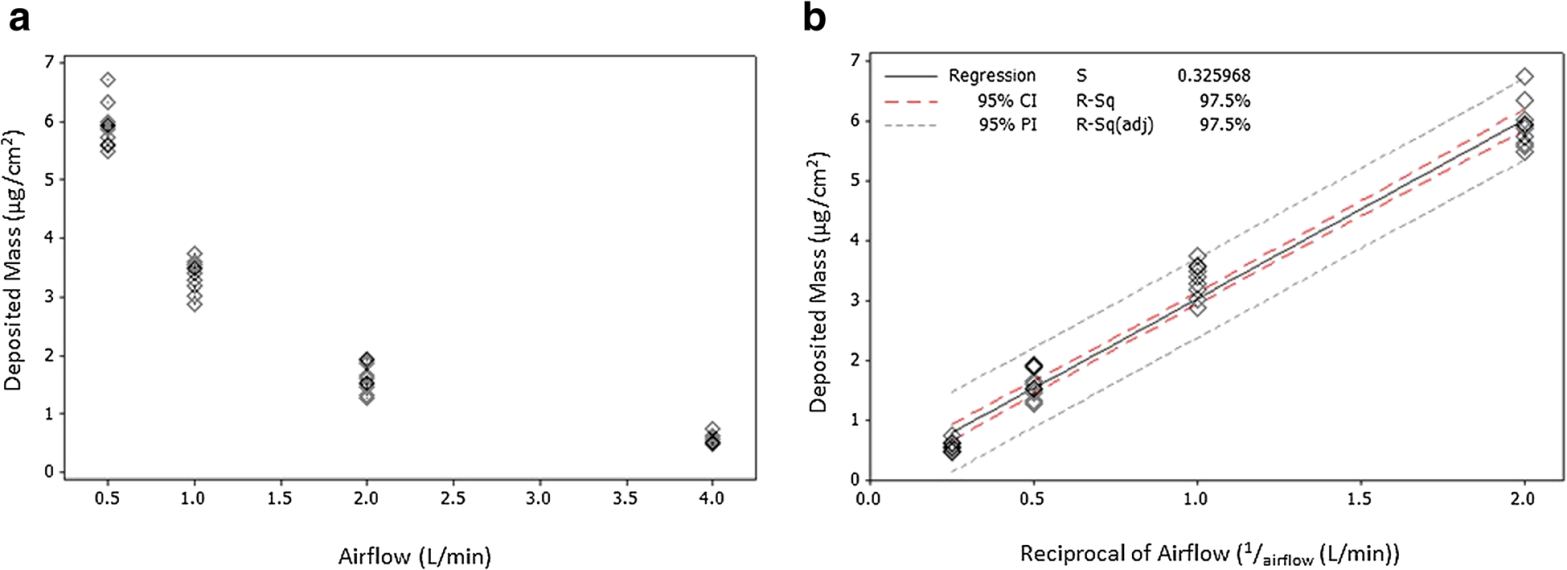
**Measurement of deposited particulate mass from a 24 minute ISO smoke exposure using 3R4F cigarettes at airflows 0.5-4.0 L/min [a] and reciprocal airflow (**^**1**^**/**_**airflow **_**(L/min)) of; 2.0, 1.0, 0.5 and 0.25 respectively [b], with a vacuum of 5 ml/min/well.** Results are based on three independent experiments with four QCM readings per experiment. **[a]** shows an individual value plot of obtained deposited mass values. For airflows of 0.5, 1.0, 2.0 and 4.0 L/min mass values of 5.9 ± 0.36, 3.3 ± 0.28, 1.6 ± 0.23 and 0.6 ± 0.08 μg/cm^2^ were obtained respectively. **[b]** shows data presented as a reciprocal of the airflow (^1^/_airflow_ (L/min)) with a regression fit correlation of R^2^ = 0.975 with 95% confidence (red dash) and probability (grey dash) intervals.

Four QCMs were installed into a Vitrocell® 6/4 CF Stainless Steel module and were used to initially assess particulate deposition at diluting airflows of 0.5, 1.0, 2.0 & 4.0 L/min across all four positions within the exposure module. The data demonstrates that there is a clear relationship between increased airflow, smoke dilution and decreased smoke particulate deposition (R^2^ = 0.975). At the highest concentration of smoke tested, which corresponds to a dilution airflow of 0.5 L/min, we were able to quantify a mean particulate deposition of 5.9 ± 0.36 μg/cm^2^ over a 24 minute exposure. For 1.0, 2.0 and 4.0 L/min airflows, the mean recorded mass was 3.3 ± 0.28, 1.6 ± 0.23 and 0.6 ± 0.08 μg/cm^2^ respectively (Figure 2).

**Figure 3:**
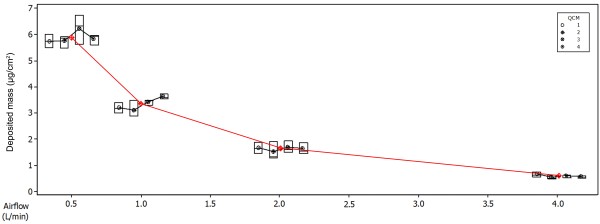
**A box plot showing deposition across the four QCM positions (1–4, left to right) within the module at airflows tested (0.5-4.0 L/min).** There were no statistical differences between QCM positions 1–4 within the exposure module at any of the airflows tested (0.5 L/min p-value 0.347, 1.0 L/min p-value 0.059, 2.0 L/min p-value 0.842, 4.0 L/min p-value 0.296). Results are based on three independent experiments.

Initial characterisation of the VC 10 using QCM technology was conducted as previously described [24] up to the 4.0 L/min airflow. However, in this study we have used QCM technology to assess deposited mass at airflows of 1.0-12.0 L/min after a 184 minute exposure (NRU) and after a 24 minute exposure (Ames), demonstrating the versatility of this tool. In addition to assessing total deposited mass across the dilution airflow range, a four QCM approach enabled the assessment of particulate deposition across the linear exposure module at all airflows tested (0.5–4.0 L/min). Although a slight ascending gradient in deposited particulate mass was observed across the module at airflows 0.5 and 1.0 L/min, no statistical difference was observed between QCM positions at any of the airflows tested (0.5 L/min p-value 0.347, 1.0 L/min p-value 0.059, 2.0 L/min p-value 0.842, 4.0 L/min p-value 0.296 - Figure 3).

### Measurement of deposited mass *in situ*

To measure deposited mass *in situ* of exposure, a single QCM unit remained installed in the final position (position 4) within the Vitrocell® exposure module (mammalian 6/4 CF and Ames). This allowed the direct monitoring of real-time particulate deposition, which gave a measure of smoke exposure conditions during *in vitro* exposure. Furthermore, this set-up enables biological data to be presented as an actual function of deposited mass obtained in real-time during exposure (Table 1).

**Table 1:** Biological and deposited mass values at all airflows tested

		**NRU**		**AMES**		
		**(184 minute exposure)**		**(24 minute exposure)**		
		**Correlation of R**^**2**^ **= 0.842**		**Correlation of R**^**2**^ **= 0.763**		
**Airflow (L/min)**	**Reciprocal of airflow (**^**1**^**/airflow (L/min))**	**Mean deposited mass for NRU exposure μg/cm**^**2**^ **± SD**	**Mean% relative cell survival ± SD**	**Mean deposited mass for Ames exposure μg/cm**^**2**^ **± SD**	**Mean revertant fold increase ± SD**	**Mean total revertants ± SD**
1.0	1.000	22.8 ± 1.7	−2.5 ± 3.3	2.30 ± 0.14	5.9 ± 1.6	78.6 ± 20.6
4.0	0.250	3.5 ± 0.1	16.7 ± 7.4	0.50 ± 0.10	4.0 ± 0.9	53.1 ± 9.6
8.0	0.125	0.8 ± 0.1	69.9 ± 13.0	0.09 ± 0.02	2.2 ± 0.4	30.2 ± 4.1
12.0	0.080	0.1 ± 0.1	96.8 ± 10.1	0.03 ± 0.01	1.6 ± 0.5	21.2 ± 5.0

Deposition data was obtained *in situ* from a 184 and 24 minute smoke exposure for NRU (Correlation R^2^ = 0.847) and the Ames assay (Correlation R^2^ = 0.763) respectively.

### Carbon monoxide

**Figure 4:**
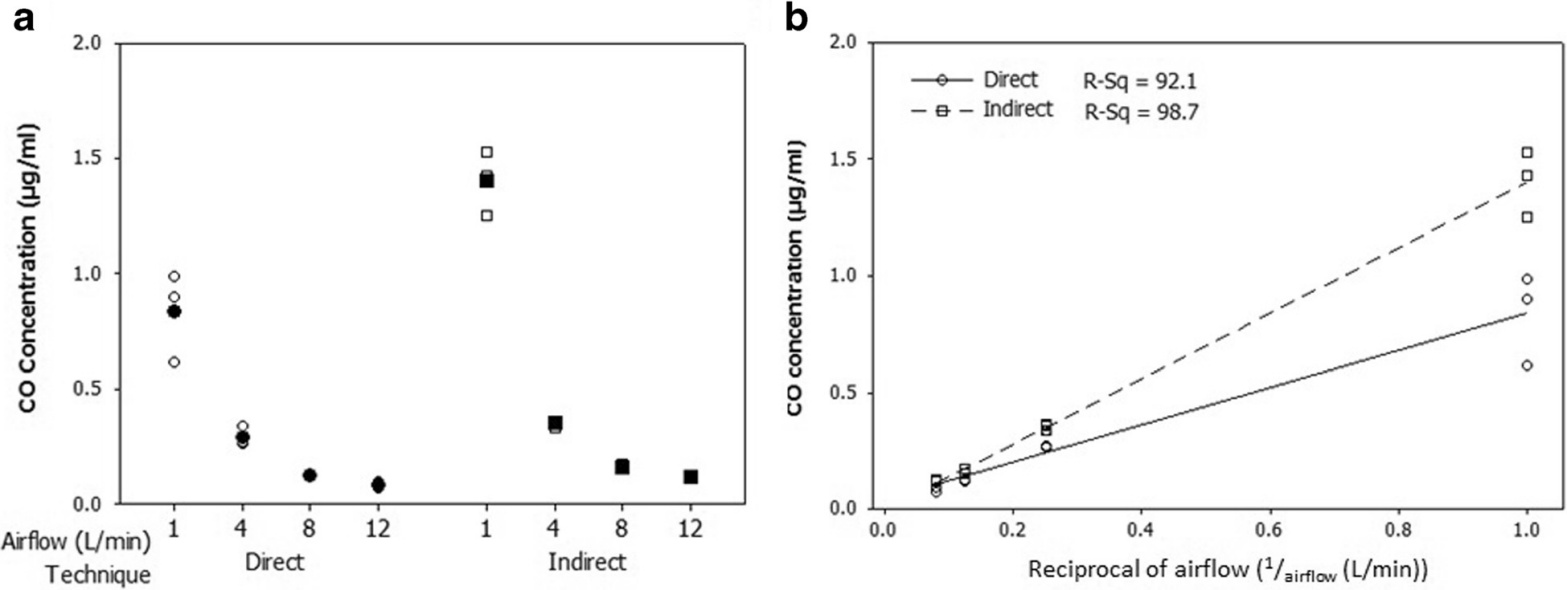
**Measurement of carbon monoxide concentrations using a ‘direct’ and ‘indirect’ technique following a 16 minute ISO smoke run using 3R4F reference cigarettes at airflows 1.0, 4.0, 8.0 and 12.0 L/min [a] and reciprocal airflow (**^**1**^**/**_**airflow **_**(L/min)) of; 1.0, 0.25, 0.125 and 0.080 respectively [b], with a vacuum of 5 ml/min/well.** Results are based on three independent experiments per airflow. **[a]** Shows an individual value plot of obtained for CO concentrations (μg/ml) and comparisons of concentrations obtained using two independent techniques, one real time and direct technique and one indirect gas-bag technique. For airflows 1.0, 4.0, 8.0 and 12.0 L/min using the direct technique CO concentrations were, 0.8 ± 0.2, 0.3 ± 0.01, 0.2 ± 0.01, and 0.1 ± 0.005 μg/ml and using the indirect technique, CO concentrations of 1.4 ± 0.1, 0.4 ± 0.04, 0.1 ± 0.01, and 0.1 ± 0.01 μg/ml were obtained respectively. **[b]** Shows data presented as a reciprocal of the airflow (^1^/_airflow_ (L/min)) with a regression fit correlation of R^2^ = 0.921 for the direct and R^2^ = 0.987 for the indirect technique.

Carbon monoxide (CO) was used as a marker to assess the vapour phase of tobacco smoke. Using a direct and indirect CO sampling method, we were able to detect CO concentration differences across the full airflow range tested (1.0-12.0 L/min). The results demonstrated a clear dose response relationship between CO and airflow (L/min), with a regression correlation of R^2^ = 0.921 and R^2^ = 0.987 for the direct and Indirect technique respectively (Figure 4).

### Neutral Red uptake

**Figure 5:**
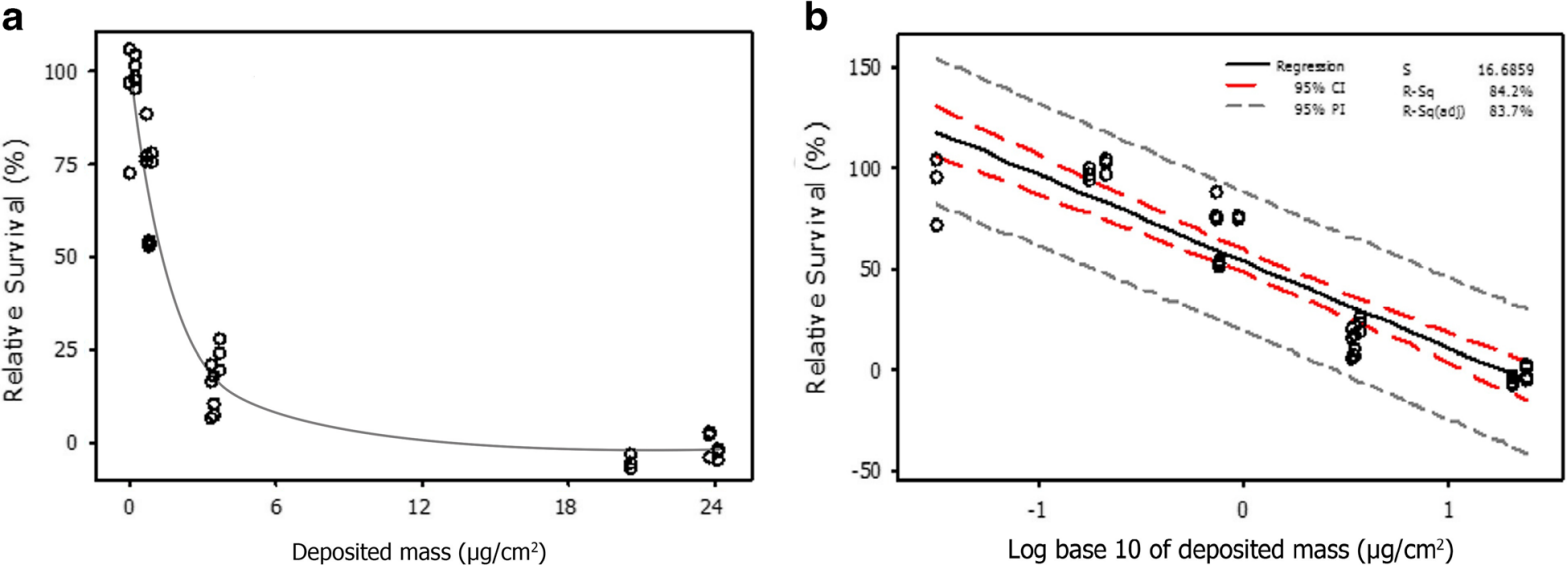
**Measurement of percentage relative survival presented as a function of deposited mass which was captured *in situ *of exposure [a] and as a Log**_**10 **_**conversion [b] following a 184 minute (23 cigarettes) 3R4F smoke exposure period. [a]** Average deposited mass for airflows 1.0, 4.0, 8.0 and 12.0 L/min were 22.8, 3.5, 0.8 and 0.1 μg/cm^2^ respectively. The calculated deposited mass IC_50_ was1.7 μg/cm^2^. **[b]** Using a Log_10_ conversion and regression analysis, the relative survival data showed a positive correlation with increased deposited mass obtained *in situ* (R^2^ = 0.84) with confidence intervals (red dash) and probability intervals (grey dash) of 95%. Results are based on three independent experiments.

The cytotoxicity of 3R4F cigarette smoke was assessed using the NRU assay across a representative range of the VC 10s dilution capability (12.0-1.0 L/min). A clear cytotoxic dose response was observed with increased smoke concentrations (12.0, 8.0, 4.0 and 1.0 L/min). The airflow ranges tested produced minimal to complete cell death. Balb/c 3 T3 cells showed no significant decrease in viability when exposed to a control airflow (air controls exposed at 0.2 L/min, 5 ml/min/well) to simulate exposure conditions. In addition to relative survival, QCM deposition data was obtained during whole smoke exposure to obtain concurrent particulate dose values. This enabled relative survival data to be presented as a function of deposited mass. For example; airflows of 12.0, 8.0, 4.0 and 1.0 L/min produced viabilities of 96.8 ± 10.1, 69.9 ± 13.0, 16.7 ± 7.4 and −2.5 ± 3.3% with corresponding deposited mass values of 0.1 ± 0.1, 0.8 ± 0.1, 3.5 ± 0.1 and 22.8 ± 1.7 μg/cm^2^ respectively. A deposited mass IC_50_ was calculated at approximately 1.7 μg/cm^2^. When data was log transformed a correlation (R^2^ = 0.84) between increased cytotoxicity and deposited mass was observed (Figure 5).

### Ames

**Figure 6:**
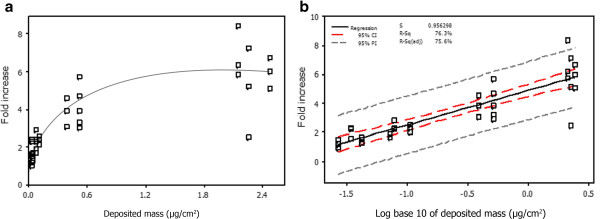
**Measurement of Ames mutation fold increases presented as a function of deposited mass which was captured *in situ *of exposure [a] and as a Log**_**10 **_**conversion [b] following a 24 minute (3 cigarettes) 3R4F smoke exposure period. [a]** Average deposited mass values for a 24 minute exposure for airflows 1.0, 4.0, 8.0 and 12.0 L/min were 2.30 ± 0.14, 0.50 ± 0.10, 0.09 ± 0.02 and 0.03 ± 0.01 μg/cm^2^ respectively. **[b]** Using a Log_10_ conversion and regression analysis fold mutation frequencies showed a positive correlation with deposited mass obtained concurrently with biological data (R^2^ = 0.763) with confidence intervals (red dash) and probability intervals (grey dash) of 95%. Results are based on three independent experiments.

Ames (YG1042) reverse mutation data correlated with increased smoke concentrations. Airflows 12.0, 8.0, 4.0 and 1.0 L/min following a 24 minutes exposure showed mean revertant counts of 21.2 ± 5.0, 30.2 ± 4.1, 53.1 ± 9.6 and 78.6 ± 20.6 respectively. In addition to mean revertants and fold increases (compared to air controls), QCM deposition data was obtained during whole smoke exposure for concurrent dose measurements. When the biological data were log transformed a correlation between fold increase in revertant (R^2^ = 0.76) colonies and deposited mass was observed (Figure 6).

## Discussion

Assessment of tobacco smoke *in vitro* has traditionally focused on the particulate phase captured on a Cambridge filter pad and eluted in DMSO [14] or bubbled through cell culture media or PBS [27]. However, these techniques do not capture the full extent of the vapour phase of cigarette smoke and semi-volatiles which not only make up the majority fraction of tobacco smoke, but include reactive chemicals with known toxicological properties [7]. Whole smoke exposure technologies exist and are gaining traction as they become more widely used, characterised and developed alongside biological end-points [21-23,28]. To ensure the full interactions of whole smoke are captured *in vitro*, we used a Vitrocell® VC 10 Smoking Robot and determined particulate deposition using QCM technology as a stand-alone characterisation tool to look at regional deposition, and also *in situ* of exposure. In addition, we have used carbon monoxide as a vapour phase marker of whole smoke and finally, produced repeatable biological dose-responses using two independent *in vitro* systems.

To measure deposited mass prior to biological exposure we used four QCMs installed into a 6/4 CF Stainless Steel Vitrocell® module and demonstrated that deposition was closely correlated with airflow (R^2^ = 0.975) with a deposited mass range of 5.9–0.36 μg/cm^2^ at a diluting airflow range of 0.5-4.0 L/min. A slight ascending concentration gradient across the exposure module was observed at airflows 0.5 and 1.0 L/min. However, in this study no statistical difference was observed between QCM positions at any of the airflows tested (0.5 L/min p-value 0.347, 1.0 L/min p-value 0.059, 2.0 L/min p-value 0.842, 4.0 L/min p-value 0.296). In addition to QCMs allowing dose to be measured in real-time *in situ* of exposure, we propose that this technology can be used as a machine QC tool, to assess dilution and deposition performance over longer periods of time. Initial deposited mass characterisation was conducted in accordance to a previously published study by Adamson et al., 2013 [24]. The results from this study show similarities between systems for total deposited mass. However, Adamson et al., 2013 [24] observed differences in the positions of the linear exposure module, whereas this study did not. This highlights the importance of understanding dilution and deposition data in each independent VC 10 system. We further utilised QCM measurements by exposing QCMs in conjunction with biological assays at all dilutions tested (1.0-12.0 L/min), demonstrating the versatility of this tool.

As whole smoke is made up of two distinct phases, it is important to characterise these phases individually. Therefore, we used CO as a vapour phase marker and characterised dilution concentrations within this set-up. Measuring CO concentrations in an *in vitro* exposure system has associated logistical challenges. For example, the CO analyser has an independent pump that pulls the diluted smoke aerosol through. Connecting this in-line can cause pressure differential problems within the system or can create a flow artefact which may affect results at low airflows. Alternatively, smoke can be captured in a Douglas bag and analysed post-exposure. This technique has the limitation that analysed smoke is artificially aged prior to analysis. In this study, we analysed CO concentrations within the system using both techniques. An in-line real-time ‘direct’ technique and an off-line post exposure ‘indirect’ technique. Both measurement techniques produced strong R^2^ correlations. However, the direct technique produced a lower correlation (R^2^ = of 0.921) compared to the indirect one (R^2^ = 0.987) and also showed a higher variation in terms of measured CO concentration again compared to the indirect technique. From a QC point of view, measuring CO using a gas bag technique is appropriate as this can be conducted outside the usual experimental conditions, or to assess changes in the system set-up. However, an in-line technique provides valuable real-time information on the exposure conditions and cigarette performance. Higher variations in the direct technique can be explained by the peaks and troughs in the CO concentrations as defined by puffing profiles, and are not present in the indirect technique as it is an homogenous mixture captured over the duration of the exposure period. Unfortunately, an indirect technique cannot be used for long exposure periods, due to the nature of gas capture in a Douglas bag. We propose the use of both techniques in combination to fully characterise the exposure system and to support *in vitro* exposure scenarios.

We also assessed the reproducibility of biological responses from two independent biological systems, using the Ames and NRU assays. Tobacco smoke produced a complete cytotoxic dose–response across the range of airflows tested (1.0-12.0 L/min) which corresponded with increased particulate deposition. The results demonstrated a deposition IC_50_ of 1.7 μg/cm^2^ for a 3 hour exposure. Moreover, the Balb/c cells were unaffected by a control airflow and were able to withstand the 3 hour exposure period with good viability, demonstrating their suitability for long term *in vitro* tobacco smoke exposure at the ALI. The Ames reverse mutation assay with strain YG1042 also demonstrated consistent biological responses, similar to that reported in a previous whole smoke study [29]. In this assay, mean revertants and fold increase in colony numbers were observed in a dose dependent manner with increasing concentrations of tobacco smoke and particulate deposition. The response from three independent experiments for both biological systems were consistent, indicating a stable exposure set-up. However, assessment of biological robustness and/or repeatability for both assays has yet to be fully assessed using the VC 10.

In this study we have presented biological data as a function of deposited mass and have defined deposited mass as the total accumulative weight deposited on the QCM crystal over the exposure period. Currently, we believe this reflects the particulate fraction of smoke with the possibility of some associated volatile or vapour phase compounds. However, the exact make up and distribution of the deposited mass fraction in this set-up has yet to be qualified or quantified and remains an area of interest. We believe that both smoke fractions are important and have distinct contributions to biological effect and It is therefore important to characterise both phases of cigarette smoke within these exposure systems.

Finally, deposited mass measurements obtained from the 6/4 CF module were different to those obtained from the Vitrocell® - Ames module at a 1.0 L/min over a 24 minute exposure. The 6/4 CF module gave a deposited mass reading of 3.3 ± 0.28 μg/cm^2^, whereas the Ames module gave 2.30 ± 0.14 μg/cm^2^. Although both supplied by Vitrocell® and designed to be used interchangeably with the VC 10 Smoking Robot, both chambers have slight variations in the width of the trumpet inside the module. The Ames module has agar-plate inserts that measure a diameter of 35 mm, whereas the 6/4CF module uses 24 mm Transwells®. To accommodate this, the trumpet circumference is larger in the Ames module compared with that of the 6/4 CF module. We propose the difference in trumpet circumference may have an impact on smoke velocities and therefore diffusion and deposition within the chamber. However, this difference was only observed at the 1.0 L/min airflow, and therefore may only be related to higher smoke concentrations that the 1.0 L/min dilution (or lower) would deliver. A more detailed study would need to be conducted to examine this observation further.

## Materials and methods

### Chemicals and reagents

All chemicals and reagents were obtained from Sigma-Aldrich (Gillingham, UK) unless otherwise stated. All tissue culture media was obtained from Gibco® via Life Technologies (Paisley, UK).

### Smoke generation

Cigarette smoke was generated using a Vitrocell® VC 10 Smoking Robot, Serial Number - VC10/090610 (Vitrocell® Systems, Waldkirch, Germany). Smoke dilutions were achieved by diluting in air (L/min), with a vacuum of 5 ml/min/well for all experiments. Flow and vacuum rates within this system were set using mass flow meters (Analyt-MTC GmbH, Mülheim, Germany) prior to experiments. For all experiments, the VC 10 smoked to the ISO smoking regime (35 ml puff over 2 seconds, once a minute). Kentucky 3R4F (9.4 mg) reference cigarettes (University of Kentucky, Kentucky, USA) were used exclusively in this study.

### Cell culture

Mouse fibroblasts (Balb/c 3 T3 clone A31) were used in the NRU assay and were obtained from the European Collection of Cell Cultures. Balb/c 3 T3 cells were maintained in Dulbecco’s Modified Eagle’s Medium (DMEM) containing 4 mM glutamine and 4.5 g/L glucose supplemented with 10% foetal calf serum and penicillin/streptomycin, at 37 ± 1°C in an atmosphere of 5% CO_2_ in air.

### Bacteria

*Salmonella typhimurium* (strain YG1042) was used in the Ames assay and obtained from the National Institute of Health Science (Tokyo, Japan). The bacterial strain YG1042 is a derivative of strain TA100 with a histidine base-pair substitution [30]. It carries an additional plasmid (pYG233) encoding for overexpression of nitroreductase and O-acetyltransferase genes. Bacteria were cultured at 37 ± 1°C for 8 hours in nutrient broth, containing Ampicillin (25 ug/ml) and Kanamycin (25 ug/ml) to obtain cells in the log phase of growth. Strain characteristic assessments were carried out according to previous reported methodologies [30-32].

### Carbon monoxide

Carbon monoxide (CO) concentrations were determined via the analysis of the diluted mainstream cigarette smoke using a Signal® 7000-FM gas analyser (Surrey, UK). Two techniques were explored, a ‘direct’ technique, where the gas analyser was attached directly to the dilution system and CO concentrations were measured in a real-time format during exposure. The ‘indirect’ technique was utilised to capture cigarette smoke in a Douglas bag (Borgwaldt, Germany) and CO concentrations were analysed post-exposure. Due to the high volume of diluting air, a 10 or 120 L Douglas bag was used as appropriate. For both techniques, two 3R4F cigarettes were smoked under ISO conditions (8 puffs per cigarette) using airflows, 1.0, 4.0, 8.0 and 12.0 L/min.

### Measurement of particulate mass

For measurement of particulate deposition within the exposure module, four QCMs (Vitrocell® Systems GmbH, Waldkirch, Germany) were installed into a 6/4 CF Stainless Steel Vitrocell® exposure module as previously described [24]. QCM technology has been incorporated into a variety of exposure chambers [20,24,26] and has been shown to correlate strongly with particulate spectrofluorescence techniques [20]. Prior to smoke exposure, the QCM module was acclimatised for several minutes prior to the baseline being set to zero. During the whole smoke generation and exposure phase, the QCM took mass readings every 2 seconds in real-time. Final deposited mass readings were only taken once the cigarette smoke had finished depositing onto the crystal, observed through a plateau phase in the real-time trace. Individual QCM positions across the linear module (1–4, distal and proximal to exhaust) were compared to assess regional deposition values across the module. In addition, data has also been presented as a function of deposited mass and as a reciprocal of airflow (^1^/_airflow_ (L/min)).

During biological exposure, three QCMs were removed from the module leaving one QCM installed in the fourth position. This allowed exposure of replicate Transwells® (NRU) or Agar plates (Ames) for biological analysis and one QCM for *in situ* measurement of particulate dose. Biological data are presented as a function of deposited mass (μg/cm^2^) obtained *in situ* of exposure.

### Neutral Red uptake

Balb/c 3 T3 cells were seeded into 24 mm Transwells® (Corning Incorporated via Fisher Scientific, UK) in 6-well plates and maintained in culture for approximately 24 hours to form a near-confluent monolayer. Cells were then exposed at the ALI to freshly generated cigarette smoke from the Vitrocell® VC 10 Smoking Robot. After exposure (184 minutes, 23 Cigarettes, 8 puffs per cigarette at airflows of 1.0, 4.0, 8.0 and 12 L/min) cells were incubated in DMEM containing 50 μg/mL Neutral Red (Sigma-Aldrich, UK) for 3 hours. Excess Neutral Red was washed away. The dye which was stored intracellularly was released by the addition of de-stain solution (ethanol: acetic acid: distilled water; (50:1:49)) and measured by absorbance at 540 nm. NRU was determined for each treatment dilution and compared to that of control cultures (air controls exposed at 0.2 L/min). Relative survival was calculated by subtracting a blank Neutral Red treated Transwell® and normalising to the air control.

### Ames

*Salmonella typhimurium* strain YG1042 was used in the presence of a 10% exogenous mammalian metabolic activation system (Aroclor 1254-induced rat liver S-9, (MolTox®, Molecular Toxicology, Inc, USA )). In brief, approximately 2x10^7^ bacterial cells were plated on to 35 mm Vogel-Bonner E agar plates in 10% S-9 buffer (prepared according to Maron and Ames [31] with 48.8 μg/mL biotin and 40 μg/mL histidine) using a spread plate methodology. Plates were then transferred to an anhydric incubator set at 37°C until dry. For smoke exposure, agar plates were transferred to the Vitrocell®-AMES module and exposed for 24 minutes (3 cigarettes, 8 puffs per cigarette) at airflows 1.0, 4.0, 8.0 and 12.0 L/min. Following exposure, plates were incubated for a further 3 days. Each plate was examined for signs of toxicity before scoring for revertant colonies (Sorcerer Image Analyser, Perceptive Instruments, Haverhill, UK).

### Statistics

All experiments were conducted on three independent occasions at airflows between 0.5-12.0 L/min with a set 5 ml/min/well vacuum. All graphs were created using MINITAB® version 16.1.0 statistical software. Statistical analysis of QCM position and deposited mass within the chamber was determined by one-way analysis of variance (ANOVA) in Minitab® 16.1.0 using Tukey’s method with a confidence level of 95.0%. Table 1 was constructed using Microsoft Excel® and show mean data ± standard deviation values for all experiments.

## Conclusions

Here we describe a study that significantly increases our working knowledge of the Vitrocell® VC 10 Smoking Robot. We conclude that in our study, under the experimental conditions tested, the VC 10 can produce stable tobacco smoke dilutions, as demonstrated by particulate deposition, measured vapour phase smoke marker delivery and biological responses from two independent *in vitro* systems. In this study biological data has been presented as a function of deposited mass obtained in real-time *in situ* of exposure, giving our biological data a gravimetric measure. We believe that this data can be better compared to others using a similar gravimetric approach irrespective of exposure system and set-up. We have not as yet addressed whether these responses can be reproduced by other VC 10 users and how variable VC 10s are from machine-to-machine and location-to-location. However, we now have the tools, techniques and applied knowledge to start addressing some of these questions.

## Abbreviations

AAI: Air-agar interface; ALI: Air-liquid interface; Ames: Ames reverse mutation assay; ANOVA: Analysis of variance; CO: Carbon monoxide; DMEM: Dulbecco’s modified eagle’s medium; DMSO: Dimethyl sulphoxide; HCI: Health Canada intense; ISO: International organisation for standardisation; NRU: Neutral red uptake assay; QC: Quality control; QCM: Quartz crystal microbalance; SD: Standard deviation; VC 10: Vitrocell® VC 10 smoking robot.

## Competing interests

The authors are employees of British American Tobacco or contracted by British American Tobacco. Covance Laboratories Ltd, Harrogate, UK, conducted all experimental work and were funded by British American Tobacco.

## Authors’ contributions

DT and JK designed the study. JK and RP conducted all experimental work. DT wrote the manuscript and analysed the data, with support from JK and JA. KS was involved in the set-up of the biological systems, whilst AD, CM and DD provided scientific support. All authors approved the final manuscript.
